# MetICA: independent component analysis for high-resolution mass-spectrometry based non-targeted metabolomics

**DOI:** 10.1186/s12859-016-0970-4

**Published:** 2016-03-02

**Authors:** Youzhong Liu, Kirill Smirnov, Marianna Lucio, Régis D. Gougeon, Hervé Alexandre, Philippe Schmitt-Kopplin

**Affiliations:** Research Unit Analytical BioGeoChemistry, Department of Environmental Sciences, Helmholtz Zentrum München, Ingolstädter Landstr.1, 85758 Neuherberg, Germany; UMR PAM Université de Bourgogne/Agrosup Dijon, Institut Universitaire de la Vigne et du Vin, Jules Guyot, Rue Claude Ladrey, BP 27877 Dijon, Cedex France; Technische Universität München, Chair of Analytical Food Chemistry, Alte Akademie 1085354, Freising-Weihenstephan, Germany

## Abstract

**Background:**

Interpreting non-targeted metabolomics data remains a challenging task. Signals from non-targeted metabolomics studies stem from a combination of biological causes, complex interactions between them and experimental bias/noise. The resulting data matrix usually contain huge number of variables and only few samples, and classical techniques using nonlinear mapping could result in computational complexity and overfitting. Independent Component Analysis (ICA) as a linear method could potentially bring more meaningful results than Principal Component Analysis (PCA). However, a major problem with most ICA algorithms is the output variations between different runs and the result of a single ICA run should be interpreted with reserve.

**Results:**

ICA was applied to simulated and experimental mass spectrometry (MS)-based non-targeted metabolomics data, under the hypothesis that underlying sources are mutually independent. Inspired from the *Icasso* algorithm, a new ICA method, *MetICA* was developed to handle the instability of ICA on complex datasets. Like the original *Icasso* algorithm*, MetICA* evaluated the algorithmic and statistical reliability of ICA runs. In addition, *MetICA* suggests two ways to select the optimal number of model components and gives an order of interpretation for the components obtained.

**Conclusions:**

Correlating the components obtained with prior biological knowledge allows understanding how non-targeted metabolomics data reflect biological nature and technical phenomena. We could also extract mass signals related to this information. This novel approach provides meaningful components due to their independent nature. Furthermore, it provides an innovative concept on which to base model selection: that of optimizing the number of reliable components instead of trying to fit the data. The current version of *MetICA* is available at https://github.com/daniellyz/MetICA.

**Electronic supplementary material:**

The online version of this article (doi:10.1186/s12859-016-0970-4) contains supplementary material, which is available to authorized users.

## Background

Metabolomics is a newly established Omics-discipline widely used in systems biology. By targeting metabolites as substrates, intermediates and products of metabolic pathways, it has been successfully applied to explain observed phenotypes [1–3] and to monitor changes in cells in response to stimuli [4, 5]. While targeted metabolomics focuses on a chosen set of metabolites [6, 7], non-targeted studies aim at the simultaneous and relative quantification of a wide breadth of metabolites in the system investigated [2, 8–11]. The latter approach demands multi-parallel analytical technology, including ultrahigh resolution mass spectrometry (MS) in direct infusion (DI) and/or linked to chromatography or electrophoresis, as well as nuclear magnetic resonance (NMR), in order to achieve complete experimental coverage [12, 13]. The spectra obtained from the different samples generated from each of these platforms are usually aligned in an intensity matrix whose rows correspond to samples and columns of overlapping chemical signals. This matrix allows the simultaneous study of mass spectra.

Previous studies have used various statistical learning methods on such data matrices to reveal differences between classes of samples and to isolate chemical signals specific to a certain class or trend [9, 13, 14]. In the context of non-targeted metabolomics, the reliability of these multivariate methods might suffer from the curse of the dimensionality problem [15]. This problem arises when datasets contain too many sparse variables (over 2000, most contain more than 10 % missing values) and very few samples (less than 100). Making a statistical model conform closely to such datasets with a limited number of training samples could result in loss of predictive power (i.e., overfitting). From another angle, since non-targeted techniques capture inegligible chemical noise and experimental bias, it may be difficult for a mathematical model to properly isolate the structure of interest [16]. Therefore applying statistical learning requires intensive method selection and validation work [8, 17–19].

Indeed, it is recommended to apply various learning algorithms in the same study to improve the reliability of the information extracted [13, 20, 21]. One common way of doing this is to use unsupervised learning (e.g., clustering, component analysis) prior to supervised methods (e.g., discriminant analysis, random forest, support vector machine), since basic data structure is revealed through simple dimension reduction, unbiased by the target information. The goal of such a non-hypothesis driven technique is to detect underlying structures relevant to the information expected, or to unnoticed subgroups, bias and noise [22]. It allows better understanding of how the non-targeted approach reflects each link of a biological experiment.

**Fig. 1:**
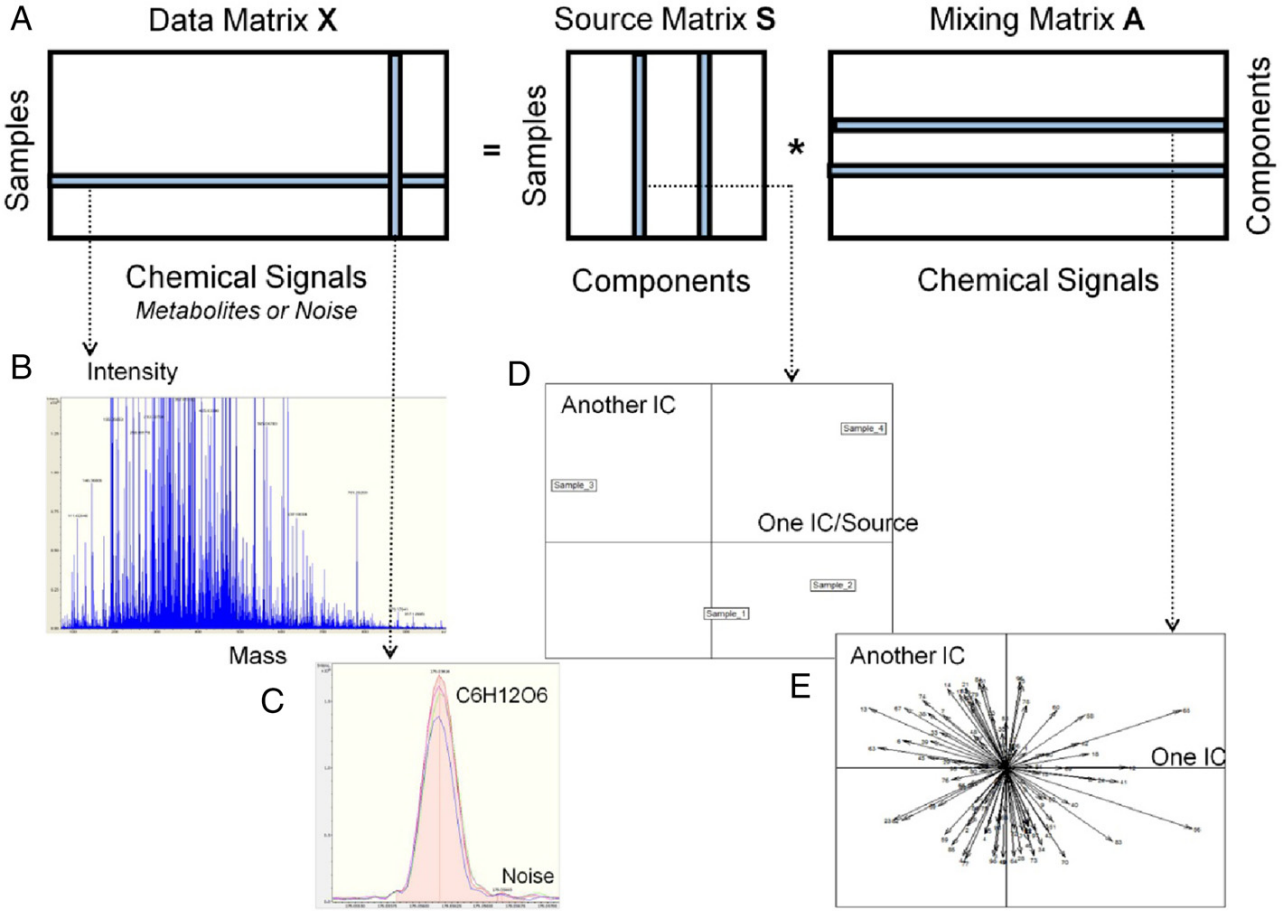
Matrix decomposition in non-targeted metabolomics. **a** ***X*** is an aligned data matrix from mass spectra of all the samples studied. The goal of ICA is to decompose *X* to a matrix *S* which contains independent sources and matrix *A* describes the linear mixture of theses source. **b** One row of ***X***: the mass spectrum of one studied sample. **c** One column of ***X***: aligned mass peaks for an annotated compound. **d** One independent source is plotted against another. The distribution of samples can be seen in the space described by these two sources. **e** represents the contribution of metabolites to these sources (loadings of metabolites)

In our study, an unsupervised learning algorithm, i.e. independent component analysis (ICA), is applied to enlarge the feature discovery in comparison to classical principal component analysis (PCA). Currently, the concept of ICA is widely used in high-dimensional data analysis such as signal processing of biomedical imaging [23, 24] and transcriptomics research [25, 26]. Recently several applications in targeted [27, 28] and low-resolution non-targeted metabolomics have achieved the goal of feature extraction [29–31] and functional investigation [7, 32]. To apply ICA we assume that the data observed *X* (*n* rows, *p* columns) are linear combinations of unknown fundamental factors or sources *S*, independent of each other (Fig. 1). Matrix *A* describes the linear combination. The sources are estimated by searching statistical components that are as independent as possible. Compared to PCA, ICA as a linear method could provide three potential benefits for non-targeted metabolomics:

- More meaningful components would be extracted by optimizing independence condition instead of variance maximization in PCA [31].
- Independence conditions detected by ICA involve both orthogonality (linear independence) and higher-order independence (e.g., exponential, polynomial), while classical PCA only ensures orthogonality between components. Therefore ICA could potentially extract additional information from the dataset.
- Since non-targeted metabolomics data usually contain huge numbers of variables and only a few samples, certain techniques using nonlinear mapping could result in computational complexity and overfitting [33]. Another drawback of such techniques is the difficulty of mapping the extracted component back in the data space. As a method based on simple linear hypothesis, ICA not only reduces the risk of overfitting but also allows the reconstruction of data in the original space.

However, major concern with ICA algorithms is stochasticity. Most ICA algorithms try to solve gradient-descent-based optimization problems such as the maximization of the non-Gaussianity of source *S* (e.g., approximated negentropy maximization in FastICA, [34]), minimization of mutual information [35, 36] and maximum likelihood estimation [37]. The randomness due to the fact that the objective function can only be optimized (maximized or minimized) locally depending on the starting point of the search (algorithm input). Thus, outputs will not be same in different runs of algorithms if the algorithm input is randomized. The curse of dimensionality makes the situation more complicated in the case of high-dimensional signal space as in non-targeted metabolomics data: it is extremely unlikely that the local minima obtained from one algorithm run will be the desired global minima and they should be interpreted with great caution.

A parameter free, Bayesian, noisy ICA algorithm has recently been developed to model the stochasticity in targeted metabolomics [7]. By applying prior distributions to *A*, *S* and noise *Γ*, Bayesian ICA estimates the posterior distribution of *S* iteratively through a mean-field-based approach [38], then *A & Γ* using a maximum a posteriori (MAP) estimator. The algorithm also suggests an optimal component selection strategy based on the Bayesian information criterion (BIC). However, tests of this algorithm on non-targeted datasets present several uncertainties: firstly, it is hard to decide on the types of priors for *A* and *Γ* in a non-targeted study since the dataset reflects the complexity of the study and has multiple manifolds; besides, the performance of the mean-field-based approach is doubtful if it cannot be compared with a full Monte Carlo sampling (too time-consuming); in addition, BIC maximization is usually impossible for high dimensional datasets with a reasonable amount of components.

Therefore we developed a heuristic method based on the FastICA algorithm and hierarchical clustering. The method, named *MetICA* is based on the *Icasso* algorithm used in medical imaging studies [39, 40]. We start with data pre-processing, including centering and dimension reduction, for which a classical PCA was used [22]. The FastICA algorithm is run many times on the PCA score matrix with *m* different inputs, generating many estimated components. Close estimates give birth to a cluster. The reliability of the FastICA algorithm can be reflected by the quality of clustering. Moreover, as with any statistical method, it is necessary to analyze the statistical reliability (significance) of the components obtained. In fact, a relatively small sample size can easily induce estimation errors [41]. Bootstrapping original datasets and examining the spread of the sources estimated might identify these uncertainties. Both reliability studies would help to decide the optimal number of components. In addition to the adaptation of the *Icasso* algorithm in non-targeted metabolomics, the novelty in the present study is the dual evaluation of algorithmic and statistical reliability for model validation. Another novelty is the automatic ordering of extracted ICs based on statistical reliability instead of only on kurtosis, as is done in other studies [7, 31]. Finally, our *MetICA* could be used for routine validation and interpretation of ICA in non-targeted metabolomics.

## Methods

### Metabolomics data acquisition and pre-treatment

Non-targeted metabolomics data were obtained from a DI-MS platform: a Bruker solariX Ion Cyclotron Resonance Fourier Transform Mass Spectrometer (ICR/FT-MS, Bruker Daltonics GmbH, Germany) equipped with a 12 Tesla superconducting magnet (Magnex Scientific Inc., UK) and an APOLO II ESI source (BrukerDaltonics GmbH, Germany) in negative ionization mode. Mass spectra of each sample were acquired with a time domain of 4 mega words over a mass range of *m/z* 100 to 1000 (Fig. 1a). The technique has ultrahigh resolution (R = 400 000 at *m/z* = 400) and high mass accuracy (0.1 ppm). After de-adduction and charge state deconvolution, mass peaks were calibrated internally according to endogenous abundant metabolites in DataAnalysis 4.1 (Bruker Daltonics GmbH, Germany) and extracted at a signal-to-noise ratio (S/N) of 4. The peaks extracted were aligned within a 1 ppm window and generated a data matrix. Each row represents the intensity of one mass signal in each sample (Fig. 1b). Masses found in less than 10 % of samples were not considered during further data analysis and other absent masses were set at zero intensity in the sample concerned. We applied the software *Netcalc* developed in-house to remove potential spectral noise and isotope peaks. This software also unambiguously annotates the elemental formula assigned to the aligned *m/z* based on a mass difference network [42]. The annotation process is considered as an unsupervised filtration that reduces data size and reveals an underlying biochemical network structure inside the data set. Our ICA algorithm is applied on this filtered data matrix.

### Biological studies

We applied the non-targeted approach followed by the ICA algorithm in a comparative study of metabolic footprinting of randomly-selected yeast strains. The goal is to detect underlying yeast phenotype subgroups based solely on their exo-metabolome in wine [43, 44]. To reach this goal, fifteen commercial *Saccharomyces* strains (S1 to S15, Lallemand Inc., France) were chosen to perform alcoholic fermentation (AF) triplicates in the same Chardonnay grape must. The strains chosen were different in species (either *S. cerevisae* or *S. bayanus*) and in origin (selected in different countries for different styles of wine or obtained by adaptive evolution) to ensure phenotype diversity. We kept the fermentation conditions consistent (e.g., volume, medium composition, temperature, etc…) between strains and replicates. At the end of AF (sugar depleted), methanolic extracts of 45 samples were studied on the ICR/FT-MS platform with the method described in the section "Metabolomics data acquisition and pre-treatment". We randomized the order of strains for the fermentation experiment and for the non-targeted study. The resulting data matrix "Yeast-Experimental.txt" (Additional file 1) had *n* = 45 rows (samples) and *p* = 2700 columns (filtered mass signals). Prior knowledge about yeast strains according to the yeast producer, including basic genetic traits, fermentation behaviors and wine characteristics, will be used for component interpretation and method validation.

### Application of *MetICA* Algorithm

**Fig. 2:**
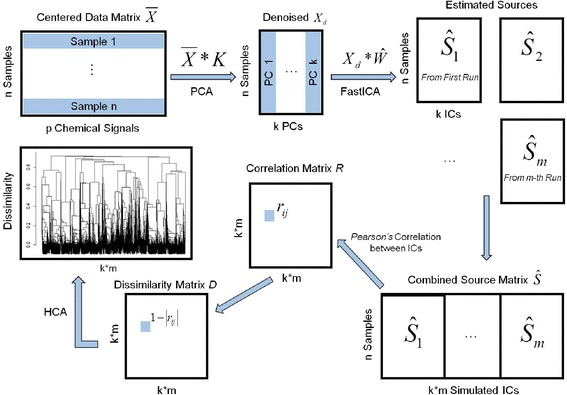
Each step of *MetICA*

We provide a concise overview of *MetICA* for non-targeted metabolomics (Fig. 2). The algorithm was mainly implemented in R version 3.1.2.

#### PCA-denoising

PCA is done by a singular value decomposition (SVD) of the centered data matrix $\overline{X}.$ The denoised matrix ${X}_d$ is obtained by ${X}_d=X*K$, where *K* is the *k* first PCs of loading matrix, obtained from the *prcomp* function in the script MetICA_fastICA.R (Additional file 1). Working on ${X}_d$ preserves 90 % of the relevant information and reduces the potential noise given by 10 % of variance.

#### FastICA algorithm

The functions *ica.R.def* ('deflation' method) and *ica.R.par* ('parallel' method) from the R package *fastICA*, version 1.2-0 (http://cran.r-project.org/web/packages/fastICA/index.html), were applied to the denoised matrix ${X}_d$ (Fig. 2 and MetICA_fastICA.R). The goal of the FastICA algorithm is to very rapidly estimate *W* or the demixing matrix. Based on a fixed-point iteration schema [34], $\widehat{W}$ is estimated to maximize the approximated negentropy under the constraint of orthogonormality. The estimated source is calculated by $\widehat{S}$ = ${X}_d$ ∗ $\widehat{\;W}$. Several rules concerning input parameters are followed while running the algorithms multiple times on ${X}_d$:

- The number of ICs is set to be the same as the number of PCs chosen for denoising.
- The hyperbolic *logcosh* function is fixed for negentropy approximation as a good general purpose contrast function [34].
- The script MetICA_fastICA.R contains two methods of extracting more than one IC: *ica.R.def* ('deflation' or one at a time) and *ica.R.par* ('parallel'). 'Deflation' avoids potential local minima [45], while 'parallel' has the power to minimize mutual information between sources [46]. Therefore each method is responsible for half of the runs.
- The matrix ${W}_0$, which is the initial point of each run, is arbitrarily sampled from a Gaussian distribution (mean = 0, variance = 1, no constraints on covariance). Other random distributions were tested and no big changes were observed for extracted components.

#### Dissimilarity matrix

The pipeline presented in Fig. 2 is achieved in MetICA_source_generator.R and MetICA_cluster_generator.R (Additional file 1). Each run of FastICA generates an estimated source matrix $\widehat{S_l}$ containing *k* components. These *k* components can be similar to a certain extent. If we combine these $\widehat{S_l}$ in a large estimated matrix $\widehat{S}$ (*n* rows, k*m columns, from function *MetICA_source_generator*), the similarity between the components from different runs can be described by *Spearman’s* correlation coefficient. In order to perform further clustering analysis, each coefficient ${r}_{ij}$ is transformed into distance or dissimilarity by ${d}_{ij}=1 - \left|{r}_{ij}\right|$ according to [47] (function *MetICA_cluster_generator*).

#### Hierarchical clustering

An agglomerative hierarchical clustering analysis (HCA) is performed on the dissimilarity matrix D with R function *hclust* (in function *MetICA_cluster_generator*). The results display a tree-like dendrogram (Fig. 2) for the hierarchical data structure: more similar components agglomerate to form a cluster and multiple clusters form a larger as a function of inter-cluster distance [48]. An average-link (AL) agglomeration method was chosen as in the original algorithm, *Icasso* [39]. Based on the hierarchical data structure, it is possible to obtain a reasonable number of clusters by cutting the dendogram at certain dissimilarity levels (*cutree* function in R). In this way, all k*m components are partitioned into a certain number of groups. Compact and well-separated clusters reveal the convergence of the FastICA algorithm. The representative points or 'centrotype' of each cluster is the point that has the minimum sum of distances to other points in the cluster (MetICA_cluster_center.R in Additional file 1). These points are considered as convergence points of FastICA and deserve further study. Therefore it is crucial to decide on the number of partitions providing the highest-quality clusters in terms of algorithmic convergence and statistical significance. Some validation strategies will be presented in the results and discussion section.

#### Production of simulated data

To confirm the power of the *MetICA* algorithm, a simulated data *SX* was generated to mimic the real non-targeted metabolomics data. The visual illustration of this process is in (Additional file 2: Figure S1) and the function used was in MetICA_simulated_generator.R (Additional file 1). From the centered yeast metabolic footprinting data $\overline{X}$, a multivariate Gaussian background noise *N* was created to have the same covariance as $\overline{X}$. In parallel, we performed a simple PCA and used non-Gaussian PCs (measured by kurtosis) to reconstruct a matrix, *RX*. The simulated *SX* is the sum of *I∗N* and *RX*, wherein *I* is a real number controlling the level of noise. The simulated data for *I* = 0.1 was stored in Yeast-Simulated.txt (Additional file 1).

## Results and discussion

### Diagnostics of simulated and experimental data

The FastICA algorithm is based on the maximization of negentropy, an exact measure of non-Gaussianity. It is equivalent to the minimization of mutual information, or searching independent components [34]. The algorithm only works when the dataset is derived from non-Gaussian sources and thus contains non-Gaussian features. Therefore we measured the non-Gaussianity of each mass using kurtosis (Additional file 2: Figure S3). The distribution of kurtosis for the experimental data showed a significant amount of super-Gaussian (kurtosis >1) and sub-Gaussian (kurtosis <−1) variables, while the background matrix *N* mainly contained Gaussian variables (kurtosis between −1 and 1). The simulated matrix *SX* contained a large number of super-Gaussian variables, knowing that two super-Gaussian PCs (PC11, kurtosis=1.9 and PC15, kurtosis=2.1) were used for generation (Additional file 2: Figure S1). Since both experimental and simulated datasets displayed non-Gaussian features, we were able to apply *MetICA* to these datasets.

### Performance of *MetICA* on simulated data

**Fig. 3:**
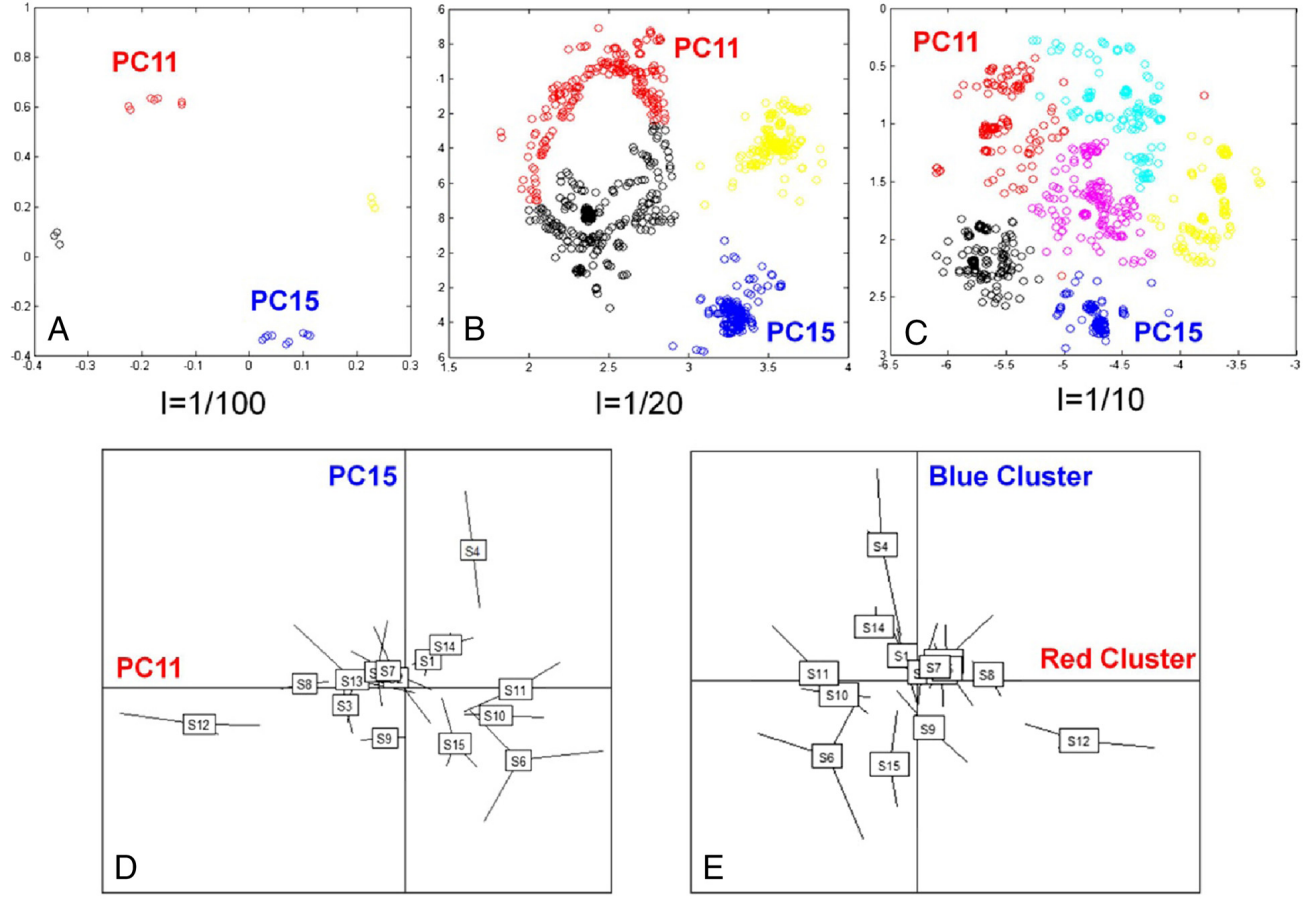
Feature extraction from simulated data. **a**, **b**, **c**, Distribution of estimated *MetICA* sources (for three background noise levels) when projected on a 2D CCA space. Sources belonging to the same hierarchical cluster have the same color. **d** The sample distribution on PC11 and PC15 used for *SX* generation: samples (top of edges) corresponding to fermentation triplicates of the same strain are connected to their gravity center (rectangle). **e** The sample distribution of the centrotypes of the red cluster and blue cluster. For any background noise level tested, the centrotypes of these two clusters carry the same strain rankings as PC11 and PC15

The *MetICA* was first tested on simulated data. The performance was evaluated based on whether the algorithm was able to retrieve the signals (PCs) used for generation. Different combinations of non-Gaussian PCs were used to generate the simulated data and evaluate the algorithm. The following is a simple example from different *SX*s generated by PC15 (${R}^2$ = 1.3 %, kurtosis = 1.9) and PC11 (${R}^2$ = 0.8 %, kurtosis = 2.1) with three levels of noise (I = 0.01, 0.05 and 0.1). We applied *MetICA* to *SX* in the way described in the previous section. The objective here was to find the optimal number of partitions for *MetICA* estimated sources. With this number, we expected to obtain high-quality clusters from HCA, with two of them representing the PCs used for generation. Our strategy started with the visualization of all the estimated sources (from different algorithm inputs) after projection onto a 2D space. A reliable projection should preserve the distance between estimated sources and hierarchical clusters should only contain neighboring points. According to our tests, Curvilinear Component Analysis (*CCA*, Matlab *SOM* Toolbox 2.0, [49]) outperformed multidimensional scaling (MDS, [48]) and the Self-Organizing Map (SOM, [50]) for this purpose. In fact, CCA preserved the distance better and gave more explicit visual separations between clusters. In order to examine the HCA results in the 2D space, the executable program MetICA_CCA.exe (Additional file 1) assigned randomly different colors to the sources belonging to different clusters. We could monitor cluster splitting by increasing the number of clusters (Additional file 2: Figure S2) until we obtained compact, well-separated clusters (Fig. 3a-c, minimal partitions necessary for different level of noise). Apart from visual monitoring, we applied a quality measure to decide the optimal number of partitions. The index is simply the ratio between the average within-clusters distance and the between-clusters distance (Additional file 2: Figure S2). The smaller the index is, the more compact and better separated the clusters seem to be on the 2D space. At the beginning this index decreases as a function of the number of clusters. From a certain point, it tends to be stable or increases, meaning that adding another cluster does not much improve the data modeling. The decision regarding the optimal number of clusters via this index is consistent with visual monitoring (Fig. 3a-c).

After the optimal number of clusters was chosen, centrotypes of clusters were verified by comparing to components used for data generation (PC11 and PC15). For all three noise levels tested, PC11 and PC15 can be described by the centrotypes of red and blue cluster, respectively (Fig. 3). In other words, *MetICA* was able to retrieve both PCs from the simulated data at different levels of noise. However, we needed 6 clusters at noise level *I* = 0.1 instead of 4 clusters at *I* = 0.05 and 3 clusters at at *I* = 0.01, proving that *MetICA* could start to extract sources from the background noise.

In brief, the performance of *MetICA* on simulated data confirmed that we could effectively study the FastICA convergence via HCA, CCA and the cluster quality index. More clusters were needed to extract underlying components when the data contained stronger noise.

### Algorithmic reliability of *MetICA* on experimental data

**Fig. 4:**
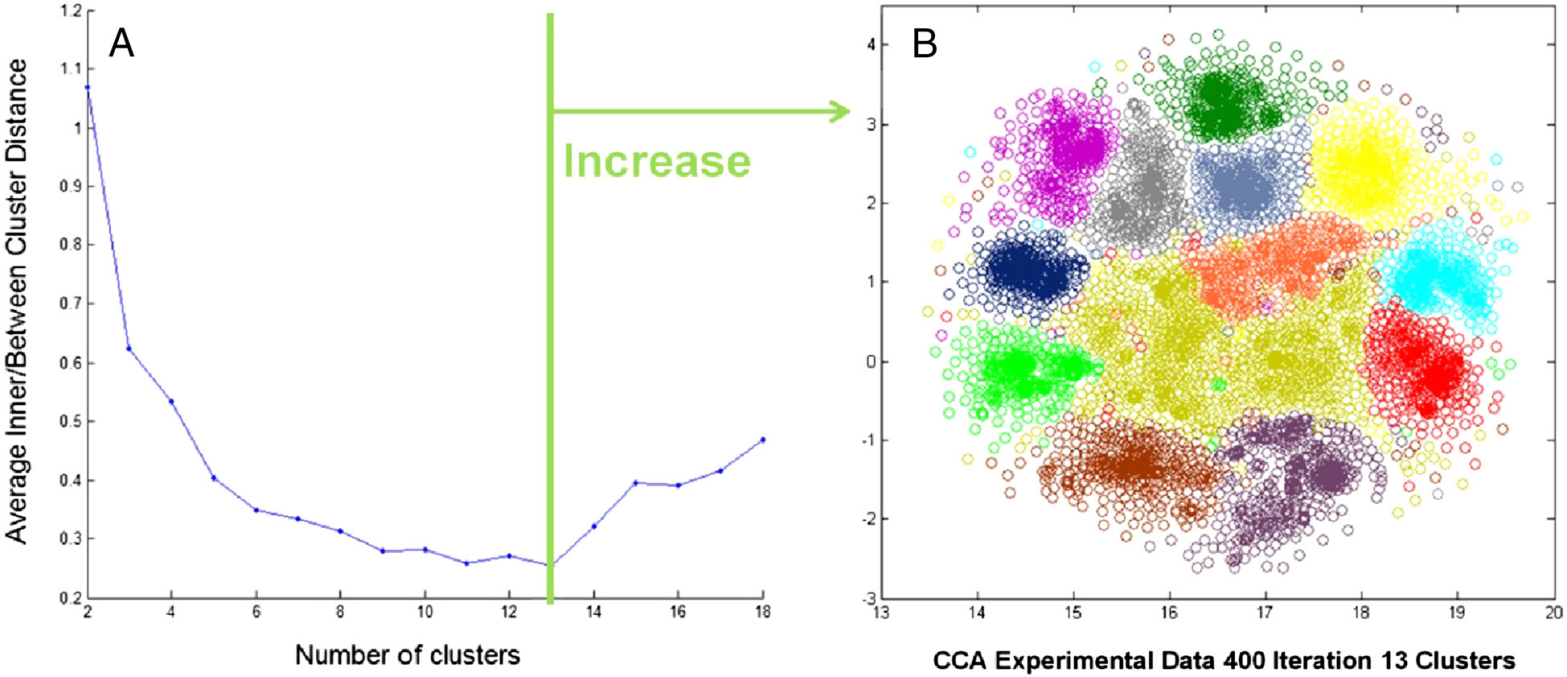
Selection of optimal cluster number. **a** The evolution of the geometric index average inner/between cluster distance as a function of number of clusters. The index is smallest at *c*=13, meaning the most compact and well-separated clusters. **b** The distribution of clusters (one color = one cluster) on the 2D space of CCA. It provides a visual confirmation for *c*

The same validation strategy was applied to the experimental data as to the simulated data. We evaluated the algorithm convergence from 15 ICs (${R}^2$ = 90.5 %) estimated in each of *m* = 800 FastICA runs. Our quality index decreased until the number of clusters reached *c* = 13 and it increased afterwards. The optimal number *c* = 13 was confirmed visually (Fig. 4). The matrix *OC* (45 * 13) contained the centrotypes of all the clusters.

### Statistical reliability of *MetICA* on experimental data

*MetICA* revealed the convergence of FastICA on non-targeted metabolomics data. However, some of the convergences observed might only haven been due to a few particular samples. Therefore it is important to evaluate the statistical significance of each centrotype obtained. However, as an unsupervised method, ICA could not be validated via prediction error since no target information could be used. Once again, as an optimization-based component analysis, cross-validation (CV) methods widely used in PCA validation [51] are inappropriate or too time-consuming. In fact, to start each CV run, datasets must be divided into two groups and the whole *MetICA* procedure has to be run on one of them (training subset). Accordingly it is necessary to validate the convergence for each CV run.

Therefore we instead applied a sophisticated bootstrapping validation. Bootstrapping means random sampling with replacement. In general, bootstrapping is considered as a slight modification of the dataset without changing its size. Bootstrapping validation is widely used for model selection in Machine Learning problems [52–54], especially when strict mathematical formulations are not available. In our case, the statistical significance of *MetICA* components was barely evaluated mathematically. Therefore we tried to find a score that described the stability of *MetICA* components subjected to bootstrapping. It was expected that components distorted by particular samples would be very sensitive to these slight modifications, while statistically significant components were expected to remain stable. The validation was implemented in the script MetICA_bootstrap.R (Additional file 1) for yeast exo-metabolome data as follows: from the original *X* (45 * 2700) we generated *B* = 100 bootstrapped data: ${X}_1$, ${X}_2$ … ${X}_B$ by replacing 5 rows of *X* each time. Then, we fixed the algorithm input, the demixing matrix ${W}_0$ and ran FastICA once on 50 bootstrapped datasets with ’parallel’ extraction and the other 50 with 'deflation' extraction. We extracted from each bootstrapped dataset *k* estimated sources (${\widehat{S}}_{b1}$, ${\widehat{S}}_{b2}$… ${\widehat{S}}_{b1k}$) to ensure ${R}^2$ > 90 % and we did likewise in each FastICA run for the original data (to ensure ${R}^2$ > 90 %).

The 13 centrotypes ${OC}_1$, ${OC}_2$… ${OC}_{13}$ from the original dataset were compared with these k estimated sources. The most correlated source ${\widehat{S}}_{ba\prime }$ was considered to be aligned to centrotype ${OC}_a$_._ The absolute *Spearman’s* correlation coefficient ${\rho}_a$ between ${OC}_a$ and ${\widehat{S}}_{ba\prime }$ was the score of ${OC}_a$ for the particular bootstrapped data. The higher the score was, the closer the estimated source was to the centrotype. The sum of scores $H=\sum {\rho}_a$ from all the bootstrapped data was our final similarity score for centrotype ${OC}_a$. It measured how similar *MetICA* centrotypes were to estimated sources of bootstrapped data, in other words, the stability of centrotypes after bootstrapping. The math input is as follows:

$$ H={\displaystyle \sum_{b=1}^B}\underset{j=1\dots k}{ \max}\left|\rho\ \left(O{C}_{a,\ }\ {S}_{bj}\right)\right| $$

The *H* score implies the statistical reliability of centrotypes given a fixed demixing matrix ${W}_0$. However, such a score might depend on the FastICA input. Therefore the scoring is repeated with fixed bootstrapped datasets but 50 randomized ${W}_0$. Finally, for each centrotype, we obtained a distribution of *H*. We used the median $\widehat{H}$ of the distribution as an exact score of the centrotype. The dispersity shows how trustworthy the score estimate is. Our empirical experiment showed that the distribution was quite weakly dispersed (Fig. 6, the results on the other datasets are similar). The visual illustration of the whole scoring process is in (Additional file 2: Figure S4).

**Fig. 5:**
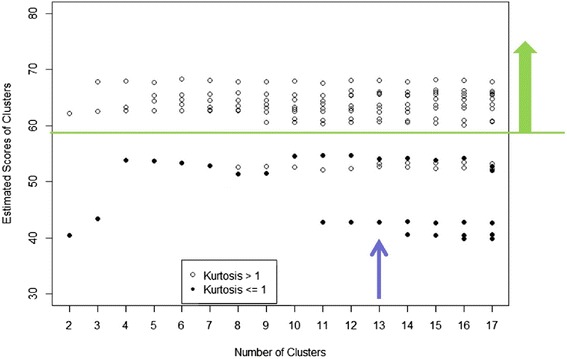
Bootstrap scores as a function of cluster number. When the cluster number is fixed, we could compute the $\widehat{H}$ score (the median of the *H* estimate) for each centrotype. Then we monitored the distribution of scores as a function of cluster number

The centrotype scoring leads to another possibility for deciding on the number of clusters. After the number of clusters was determined, we could evaluate the $\widehat{H}$ of each centrotype after which we obtained a score distribution of all the centrotypes for the particular number of clusters. Therefore we could monitor the $\widehat{H}$ for all the centrotypes as a function of the number of clusters (Fig. 5) and select the optimal number based on the amount of centrotypes containing a higher $\widehat{H}$. We observed a pattern of statistically reliable super-Gaussian centrotypes ($\widehat{H}>58$, points above the green line in Fig. 5). At *c = 13* clusters suggested previously by the quality index, we obtained 9 such centrotypes. Low significant centrotypes seemed to occur when we further increased the number of clusters, which means that *c = 13* was also a good decision in terms of statistical reliability.

**Fig. 6:**
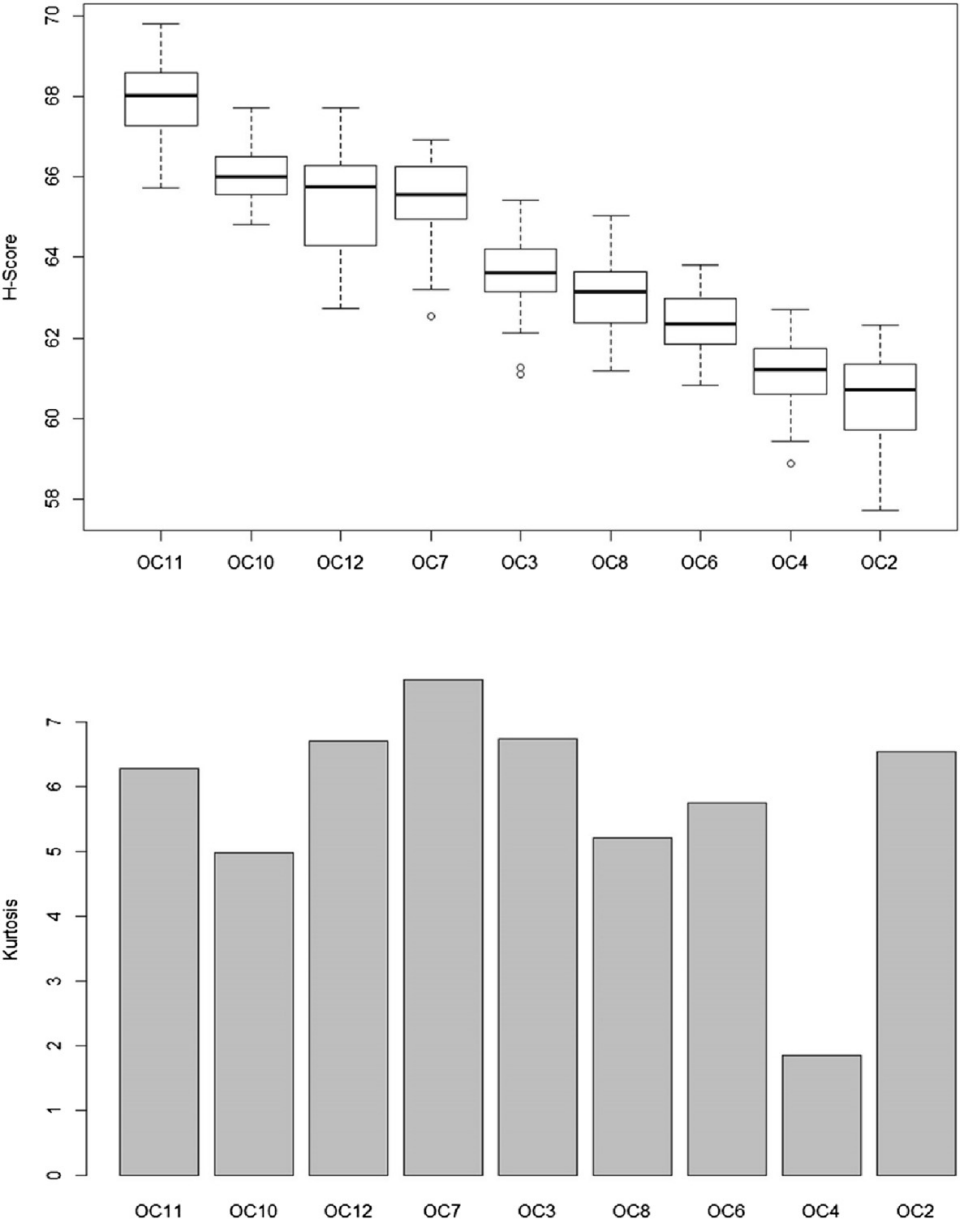
*H* estimates and kurtosis of centrotypes. The upper figures shows the distribution of the *H* estimate of each centrotype by box plot, sorted by their median, e.g. ${OC}_{11}$ has the highest $\widehat{H}$ so it is considered to be the most statistically-reliable. The lower figure shows the kurtosis of each corresponding centrotype

Afterwards a comparison was made between the bootstrap score and kurtosis of these centrotypes. In previous studies, super-Gaussian distributed components usually indicated interesting class separation structures while Gaussian-like distribution (kurtosis close to 0) or sub-Gaussian (kurtosis < −1) contained less information [31]. In Fig. 5, it can be seen that low kurtosis centrotypes also have a low $\widehat{H}$. However, the highest kurtosis does not ensure the highest bootstrap score (Fig. 6).

### Component order and interpretation

The components extracted by a single ICA run have no order. However, we give an interpretation order for the centrotypes obtained based on their bootstrap score $\widehat{H}$. We first interpret the centrotypes that have relatively higher $\widehat{H}$ (statistically significant) with smaller error bars (stable after changing algorithm inputs). The following are biological interpretations for some of the top nine centrotypes (Fig. 6). The script for visualization of scores and loadings is in Tutorial.pdf (Additional file 1).

#### ICA detects outliers

ICA seems to be sensitive to outliers. For instance, sample R1S6 (wine fermented by strain S6 in the first replicate) has an extreme negative score on ${OC}_6$ compared to the other samples, including the two other replicates of S6 (Additional file 2: Figure S5A). The same situation was also observed on ${OC}_2$ & ${OC}_3$ (Additional file 2: Figure S5B-C). Although the interpretation of these outliers is not so obvious, the reliability of the centrotypes encouraged us to investigate the potential technical errors.

#### ICA detects phenotype separations

**Fig. 7:**
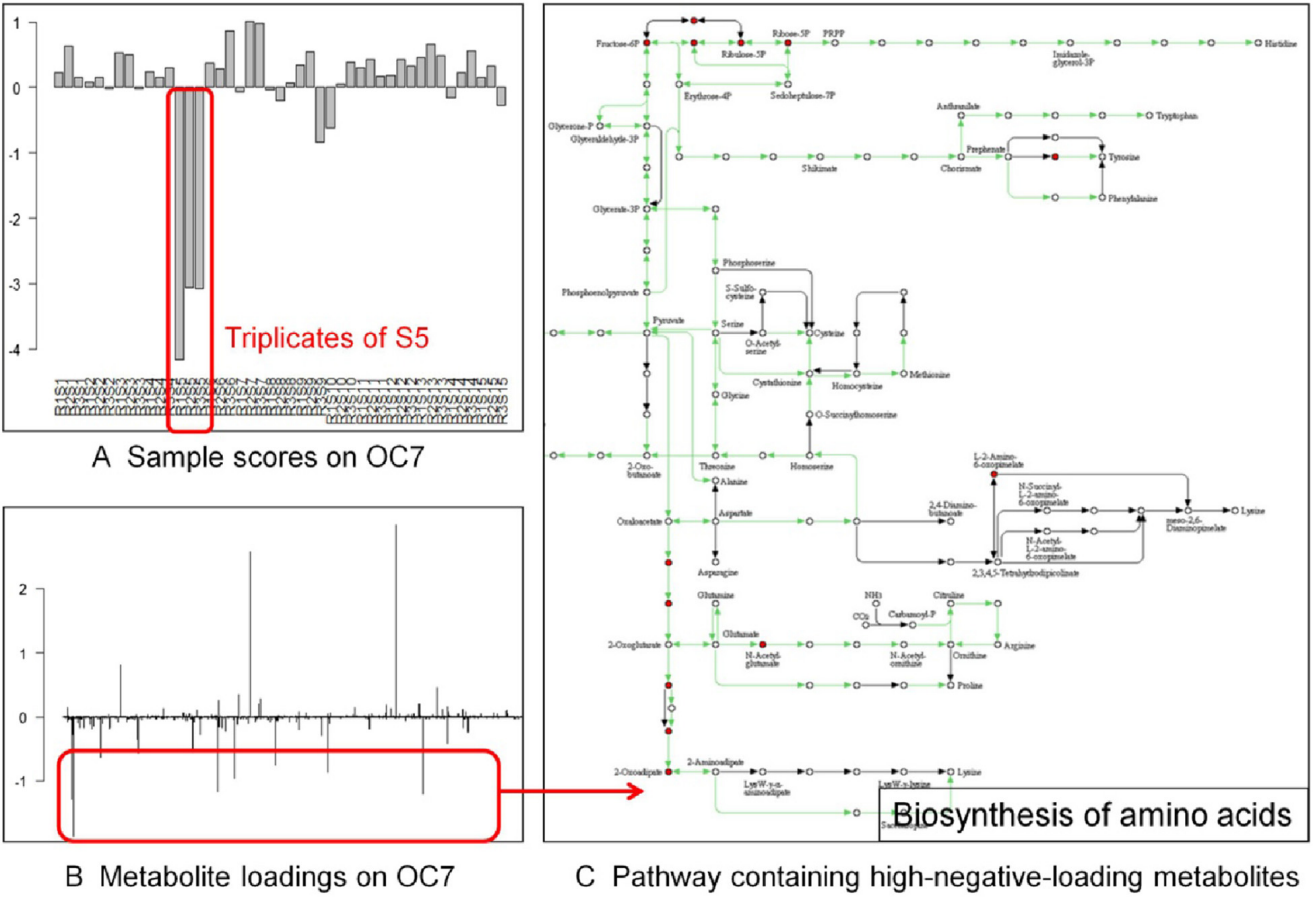
Interpretation of a centrotype. **a** The score of each sample on ${OC}_7$. The three wines from the fermentation triplicates of strain S5 (R1S5, R2S5, R3S5) all have higher negative scores. **b** Loadings of metabolites on ${OC}_7$. Metabolite having higher negative loadings contribute to the separation of S5 from other strains. **c** Many of these metabolites are annotated in the biosynthesis of amino acids. Here, red nodes are annotated compounds

The three samples (wines from fermentation triplicates) of strain S5 have higher negative scores than all the other samples on ${OC}_7$ (Fig. 7). In general, if one component carries biological information, it is interesting to know which mass signals are highly involved. These signals have higher loadings in weights matrix *A,* which is the pseudo-inverse of the product of whitening matrix *K* and demixing matrix *W*:

$$ A={(KW)}^t{\left(KW{(KW)}^t\right)}^{-1} $$

Mass signals with the top 100 highest negative loadings on ${OC}_7$ were extracted. The concentration of these metabolites should be higher in wines fermented by S5 than other strains. Under the assumption that exo-metabolome reflects cell activity, we mapped the extracted mass signals from the yeast metabolic network using the MassTRIX server (http://masstrix3.helmholtz-muenchen.de/masstrix3/) [55]. Among 49 annotated masses, 13 were metabolites in the yeast metabolic pathway biosynthesis of amino acids (Fig. 7). This observation was in accordance with information from the yeast provider: strain S5 could synthesize more amino acids and thus stimulate secondary fermentation in wine.

Similar results were observed on ${OC}_{10}$: triplicates of S3 (commercial name: ECA5) had much higher positive scores than the other samples (Additional file 2: Figure S5D). Corresponding metabolites annotated on MassTRIX revealed enrichment in several pathways in central carbon metabolism, such as fructose & mannose metabolism, the Pentose phosphate pathway and the TCA cycle. In fact, ECA5 is a strain created by adaptive evolution to enhance sugar metabolism, notably the metabolic flux in the Pentose phosphate pathway [56].

### Comparison to other ICA algorithms

The performance of *MetICA* was compared to other ICA algorithms (Table 1) using another non-targeted ICR/FT-MS-based metabolomics dataset (published data [57]). The data matrix counted initially 18591 signals measured in 51 urine samples from doped athletes, clean athletes and volunteers (non-athletes). For the purpose of filtering and formula annotation, such high data dimension was more efficiently handled by our in-house developed software *Netcalc* compared to other standard approaches, such as *ChemoSpec* (http://cran.r-project.org/web/packages/ChemoSpec/index.html) and *MetaboAnalyst* (http://www.metaboanalyst.ca/). The reduced data matrix Doping.txt (Additional file 1) with 9279 mass signals remained were analyzed directly with *MetICA*, as well as two FastICA algorithms in R (‘Parallel’ and ‘Deflation’). Four other ICA packages were tested on the PCA score matrix *Xd* (51 rows, 43 columns, ordered by variance explained): *icapca* in R [58], *icamix* in R (http://cran.r-project.org/web/packages/icamix/), *kernel-ica* toolbox version 1.2 in Matlab with a Gaussian kernel [59] and *mean field ICA* toolbox in Matlab for Bayesian ICA described previously [7]. If ‘out of memory’ problem occurred or the simulation failed to produce reasonable results, the corresponding package was applied only on first few columns of *Xd* (variance explained was reduced, Table 1 [1, 2]). For all 7 ICA methods tested, 10 replicates were made with randomized algorithm inputs. We evaluated the shapes of extracted components Table 1 [3–5]), the stability between simulation runs (Table 1 [6]) and the reliability of components & model (Table 1 [7, 8]).

**Table 1:** Comparison between different ICA algorithms

	MetICA	FastICA	FastICA	icapca	icamix	kernel-ICA	Bayesian
	18 Clusters	'Parallel'	'Deflation'			Gaussian	
[1] Variance Explained	90 %	90 %	90 %	75.7 %	99 %	99 %	99 %
[2] Component Extracted	18	20	20	7	43	43	9
[3] Maximal Kurtosis	44.1	43.9	44.1	44.1	43.6	3.9	29.8
[4] Minimal abs(Kurtosis)	1.6	3.4	1.9	0.5	15.1	0.008	0.007
[5] Minimal Kurtosis	−1.6	3.4	−2	0.5	15.1	−1.7	−0.9
[6] Stable Components	12/18	20/20	9/20	3/7	43/43	0/43	1/9
[7] Model Selection	HCA	-	-	LOO-CV	Likelihood	-	BIC
[8] Component Order	Bootstrap	-	Deflation	Variance	-	Deflation	Kurtosis

Seven ICA algorithms were compared based on [1] maximal percentage of variance the algorithm could handle (depending on the computer memory), [2] optimal number of components that the algorithm suggests, [3] kurtosis of the most super-Gaussian component [4] kurtosis of the most Gaussian component, [5] minimal kurtosis of components (the most sub-Gaussian when it is negative), [6] number of consistent components extracted in all 10 algorithms runs with an absolute *Spearman's* correlation between them higher than 0.8 and on whether the algorithm suggests [7] model selection criteria [8] importance order of components

The comparison revealed that *MetICA* extracted both super-Gaussian and sub-Gaussian components, while 'parallel' FastICA, *icapca* and *icamix* only highlighted super-Gaussian signals. Components from Kernel-ICA & Bayesian-ICA were more Gaussian-distributed. Among seven algorithms, 'parallel' FastICA and *icamix* gave consistent results between simulation runs. *MetICA* resulted in 12 out of 18 stable components if we fixed the number of clusters at 18. Our studies also showed that the amount of stable components would increase if the cluster number was tuned for each run through cluster visualization or bootstrapping. In the end, *MetICA* was among the few algorithms that suggested both model selection and component ranking. The *icapca* package suggests a reliable LOO-CV-based component selection, but the simulation seemed computationally intensive for our dataset. As a result, the model from *icapca* only explained 75.7 % of total variance.

## Conclusion

In this paper, we developed the *MetICA* routine for the application and validation of ICA on non-targeted metabolomics data. We adapted *Icasso*, an algorithm previously used in medical signal processing, to our MS-based yeast exo-metabolome data. We studied the convergence of FastICA in a way slightly different from that in the original *Icasso* version [31]: *Spearman’s* correlation was used instead of *Pearson’s* correlation to simplify the relations between estimated sources; the cluster number was selected based on a simple geometric index on projected space, instead of quantitative indices in the original space. These two simplifications improved the efficiency for high-dimensional data, since we tried to keep the maximum variance after PCA-denoising while having enough FastICA runs. As a result, we usually generate a huge amount of estimated components (>5000), but using the original *Icasso* is too time-consuming to handle this amount. An alternative fast approach for estimated sources clustering was to use the rounded kurtosis value [60]. However, *MetICA* seems to be much more sensitive to detect non-similarities for non-targeted metabolomics data.

Furthermore, we investigated the statistical reliability of convergence points by comparing them to FastICA estimates for bootstrapped data. Reliable centrotypes revealed strong phenotype separations and pathway differences between phenotypes.

From the modeling viewpoint, Bayesian ICA optimized the model by BIC - a trade-off between likelihood (how much the model fits the data) and the risk of over-fitting. When processing high dimension data became difficult, our method provided an alternative mean of model optimization: increasing the number of reliable components instead of fitting the data. We suggested two ways of deciding the optimal number of model components, namely the number of clusters: either by using a cluster quality index (algorithmic reliability), or through the bootstrap scores of all the centrotypes (statistical reliability).

The whole *MetICA* routine was tested on simulated data and several MS-based non-targeted metabolomics data, including low resolution MS datasets (an example is provided in Additional file 3). Compared to other ICA methods, *MetICA* could efficiently decide a reasonable number of clusters based on algorithmic reliability. The bootstrap scores further validated this decision. For both high and low mass resolution and for any biological matrices, *MetICA* was able to handle more than 10 000 features and to sensitively select reliable models.

Since our routine was based on a simple linear model, we could easily reconstruct the original dataset and calculate the fitting error. Therefore, our procedure could also be further used for dimension reduction before applying supervised statistical methods, or data denoising to remove undesirable signals (bias and instrumental noise) [61]. All in all, it opens a door for extracting non-Gaussian information and non-linear independence from non-targeted metabolomics data.

## Additional files

Additional file 1: Source code and raw datasets used for *MetICA* evaluation. Source code, raw datasets and user manual were also available at https://github.com/daniellyz/MetICA. (ZIP 4725 kb) Additional file 2: Figure S1. Generation of simulated data. The simulated data *SX* was generated by adding the background noise ***N*** (multivariate Gaussian distribution derived from original data) to a matrix reconstructed by two selected non-Gaussian PCs (PC11 & 15). The blue intensity here represents signal intensity. Figure S2. Hierarchical clusters in 2D space. Distribution of estimated *MetICA* sources from simulated data when projected on a 2D CCA space. Sources belonging to the same hierarchical cluster have the same color. The splitting of the dark blue cluster into black, dark blue and cyan clusters was seen when we increased the cluster number NC from 2 to 4. It splitted again when NC increased to 6. The quality index is the ratio between the average within-cluster distance (R1, the distance between the estimate and the cluster center it belongs to) and the average between-cluster distance (R2, the distance between each cluster center to the global center of all estimates). Figure S3. Kurtosis distribution of all variables (masses). Three histograms represent kurtosis distributions for experimental data *X_exp*, simulated background noise *N* and simulated data *SX* (I=0.01), respectively. Figure S4. Illustration for bootstrap scores. For a fixed algorithm input, FastICA runs on *B* different bootstrapped data. The centrotype ${OC}_a$ (blue) is compared to all the estimated sources from each run. The *Spearman* correlation coefficient (red) to the most correlated estimate (green) is the similarity score we are seeking. The final score ${H}_{OCa}$ is the sum of scores from all the bootstrapped data. Figure S5. Scores of samples on some centrotypes. A) On ${OC}_6$, sample R1S6 (wine fermented by strain S6 in the first replicate) has an extreme negative score, so it is considered as an outlier. B) C) For the same reason as R1S6 on ${OC}_6$, samples R3S6, R2S4 and R3S11 are considered as outliers. D) The three wines from the fermentation triplicates of strain S3 (R1S3, R2S3 and R3S3) all have higher positive scores. (DOCX 996 kb) Additional file 3: Evaluation of *MetICA* on lower resolution metabolomic data. Additional text and figures are provided to illustrate the application of MetICA on lower resolution LC-MS data. (DOCX 280 kb)

## Abbreviations

AF: alcoholic fermentation
AL: average-link
BIC: Bayesian information criterion
CCA: curvilinear component analysis
CV: cross-validation
DI: direct infusion
HCA: hierarchical clustering analysis
ICA: independent component analysis
ICR/FT-MS: ion cyclotron resonance Fourier transform mass spectrometer
LOO-CV: leave-one-out cross-validation
MAP: maximum a posteriori
MDS: multidimensional scaling
MS: mass spectrometry
NMR: nuclear magnetic resonance
PCA: principal component analysis
SOM: self-organizing map
