# Supplementary material for: MetICA: independent component analysis for high-resolution mass-spectrometry based non-targeted metabolomics
Source: BMC Bioinformatics. 2016 Mar 2;17:114. doi: 10.1186/s12859-016-0970-4 (PMC4776428; doi:10.1186/s12859-016-0970-4)
Supplement: Additional file 1: — Source code and raw datasets used for MetICA evaluation. Source code, raw datasets and user manual were also available at https://github.com/daniellyz/MetICA. (ZIP 4725 kb) [file 12859_2016_970_MOESM1_ESM.zip › MetICA/Tutorial.pdf]

Manual of MetICA

Example of application on yeast exo-metabolomics data

Step 1. (Please type in R console) Initialization of MetICA

```
source('MetICA_load_all.R')
```

Step 2. (Please type in R console) Load and center data

```
new_data=data.matrix(read.table('Yeast-Experimental.txt',sep='\t',dec='.',header=T,check.names=FALSE))

new_data=new_data[2:nrow(new_data),2:ncol(new_data)]

row.names(new_data)=read.table('Yeast-Experimental.txt',sep='\t',dec='.',header=T)[2:(nrow(new_data)+1),1]

new_data_centered=scale(new_data,scale=F)
```

Step 3. (Please type in R console) Estimation of sources from the whole training dataset with 800 random inputs, 90% of variance was kept:

```
M1=MetICA_source_generator(new_data_centered,0.9,'gaussian',800)
```

Step 4. (Please type in R console) Clustering of estimated sources. This steps generates three .txt files 'source_list.txt', 'cluster_labels.txt' and 'distance.txt'.

```
M2=MetICA_cluster_generator(M1$S,'spearman',M1$IC)
```

Step 5. Please use the MetICA_CCA graphical interface to visualize clusters. Please make sure that the MCRInstaller (http://www.mathworks.com/products/compiler/mcr/) is installed. The executable application MetICA_CCA.exe can be found in the folder 'for_testing'.

a) Please find the 3 .txt files generated by Step 4 and load them successively into the application:

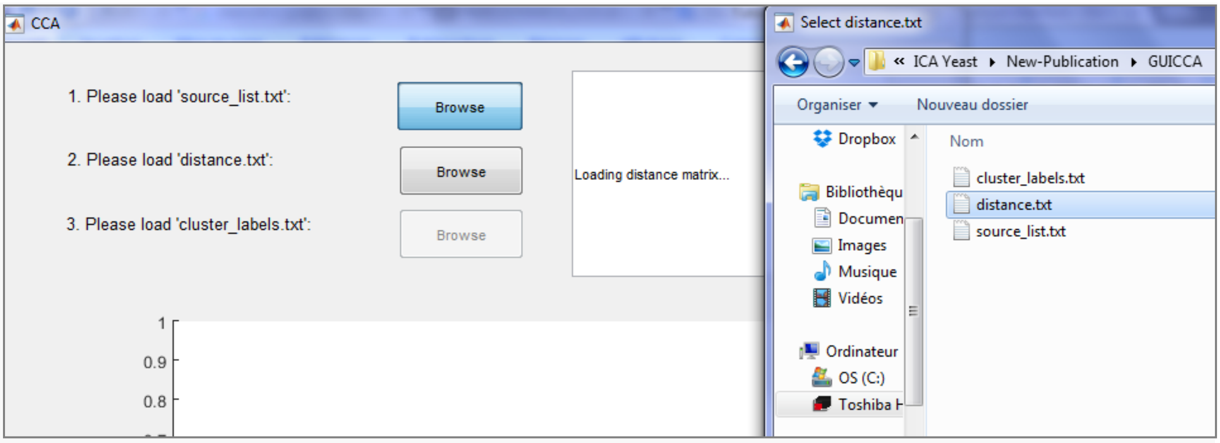

b) Once the three files were loaded, please click on 'start' to perform CCA (curvilinear component analysis). This analysis could project data on a 2D space. The default number of iterations is 200.

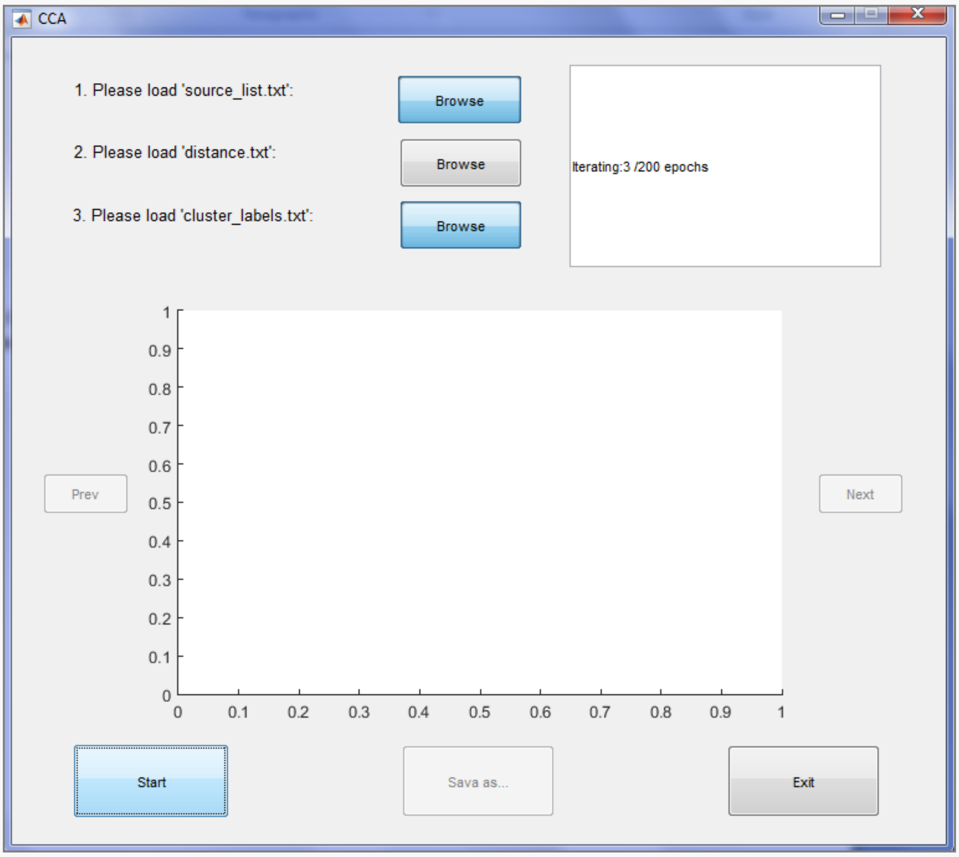

C) When CCA simulation is finished, users could click on 'Next' to visualize the location of estimated sources on the 2D space. The sources that belong to different clusters were assigned random colors. Please click 'Next' to increase the number of clusters or 'Prev' to decrease. Please monitor carefully the cluster splitting until we found the optimal number of clusters (the situation where we have compact and well-separated clusters)

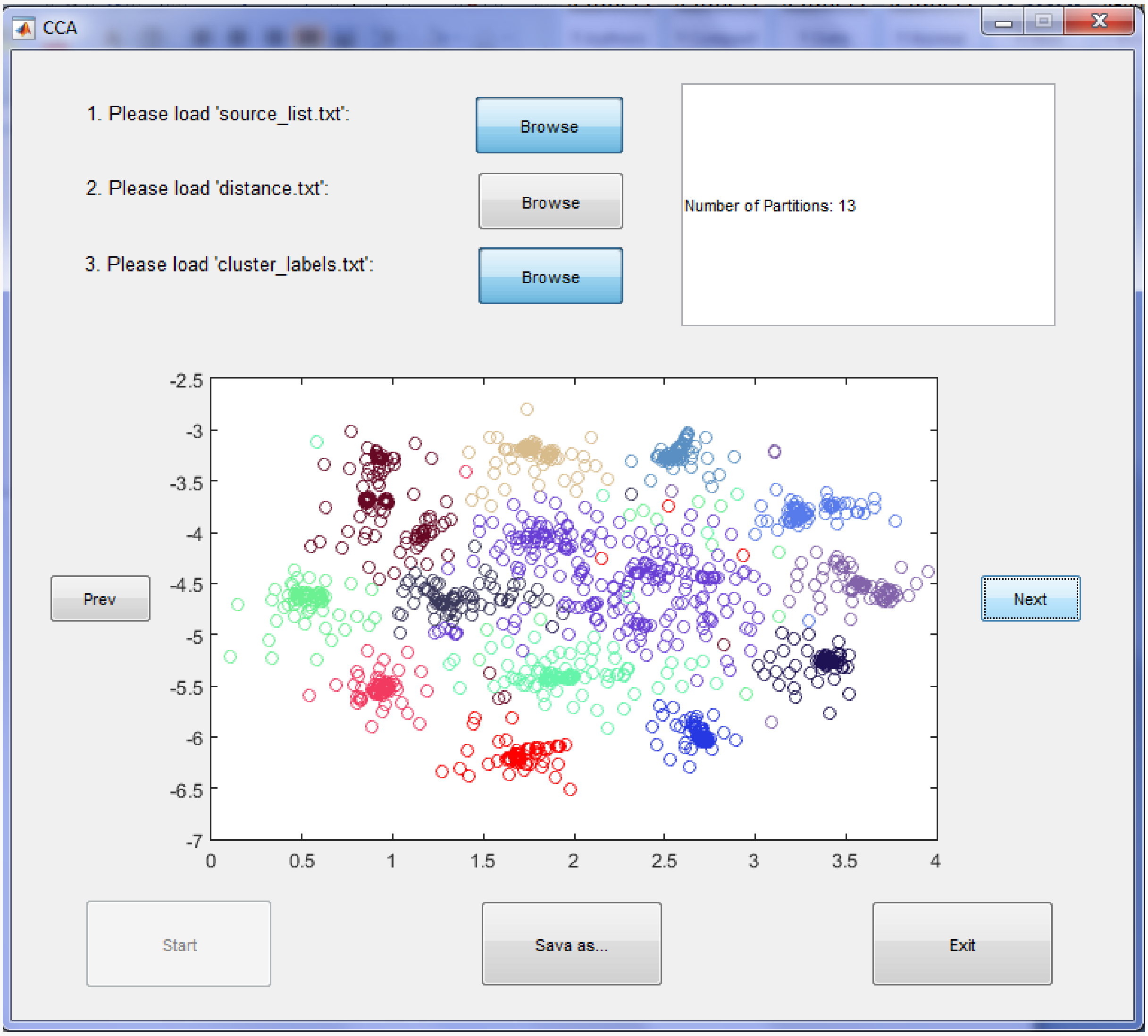

Step 6. (Please type in R console) When 13 was chosen as the optimal number of clusters according to step 5, we could compute the center (centrotype) of each cluster. Each column of the output matrix M3$center corresponds to one centrotype. User could interpret these centrotypes in the same way as PCA scores.

```
M3=MetICA_cluster_center(M2$S,M2$D,19)

cscores=M1$S[,M3$center_ID]

barplot(cscores[,2],las=2) # Score plot for OC2
```

Step 7. (Please type in R console) This step ranks the centrotypes according to the statistical reliability. A score and the variation of the score were calculated based on bootstrapped datasets.

```
M4=MetICA_bootstrap(new_data_centered,5,M3$center,0.9,'gaussian',100,50)
```
