# Supplementary material for: MetICA: independent component analysis for high-resolution mass-spectrometry based non-targeted metabolomics
Source: BMC Bioinformatics. 2016 Mar 2;17:114. doi: 10.1186/s12859-016-0970-4 (PMC4776428; doi:10.1186/s12859-016-0970-4)
Supplement: Additional file 2: — Figure S1. Generation of simulated data. The simulated data SX was generated by adding the background noise N (multivariate Gaussian distribution derived from original data) to a matrix reconstructed by two selected non-Gaussian PCs (PC11 & 15). The blue intensity here represents signal intensity. Figure S2. Hierarchical clusters in 2D space. Distribution of estimated MetICA sources from simulated data when projected on a 2D CCA space. Sources belonging to the same hierarchical cluster have the same color. The splitting of the dark blue cluster into black, dark blue and cyan clusters was seen when we increased the cluster number NC from 2 to 4. It splitted again when NC increased to 6. The quality index is the ratio between the average within-cluster distance (R1, the distance between the estimate and the cluster center it belongs to) and the average between-cluster distance (R2, the distance between each cluster center to the global center of all estimates). Figure S3. Kurtosis distribution of all variables (masses). Three histograms represent kurtosis distributions for experimental data X_exp, simulated background noise N and simulated data SX (I=0.01), respectively. Figure S4. Illustration for bootstrap scores. For a fixed algorithm input, FastICA runs on B different bootstrapped data. The centrotype ${OC}_a$OCa (blue) is compared to all the estimated sources from each run. The Spearman correlation coefficient (red) to the most correlated estimate (green) is the similarity score we are seeking. The final score [file 12859_2016_970_MOESM2_ESM.docx]

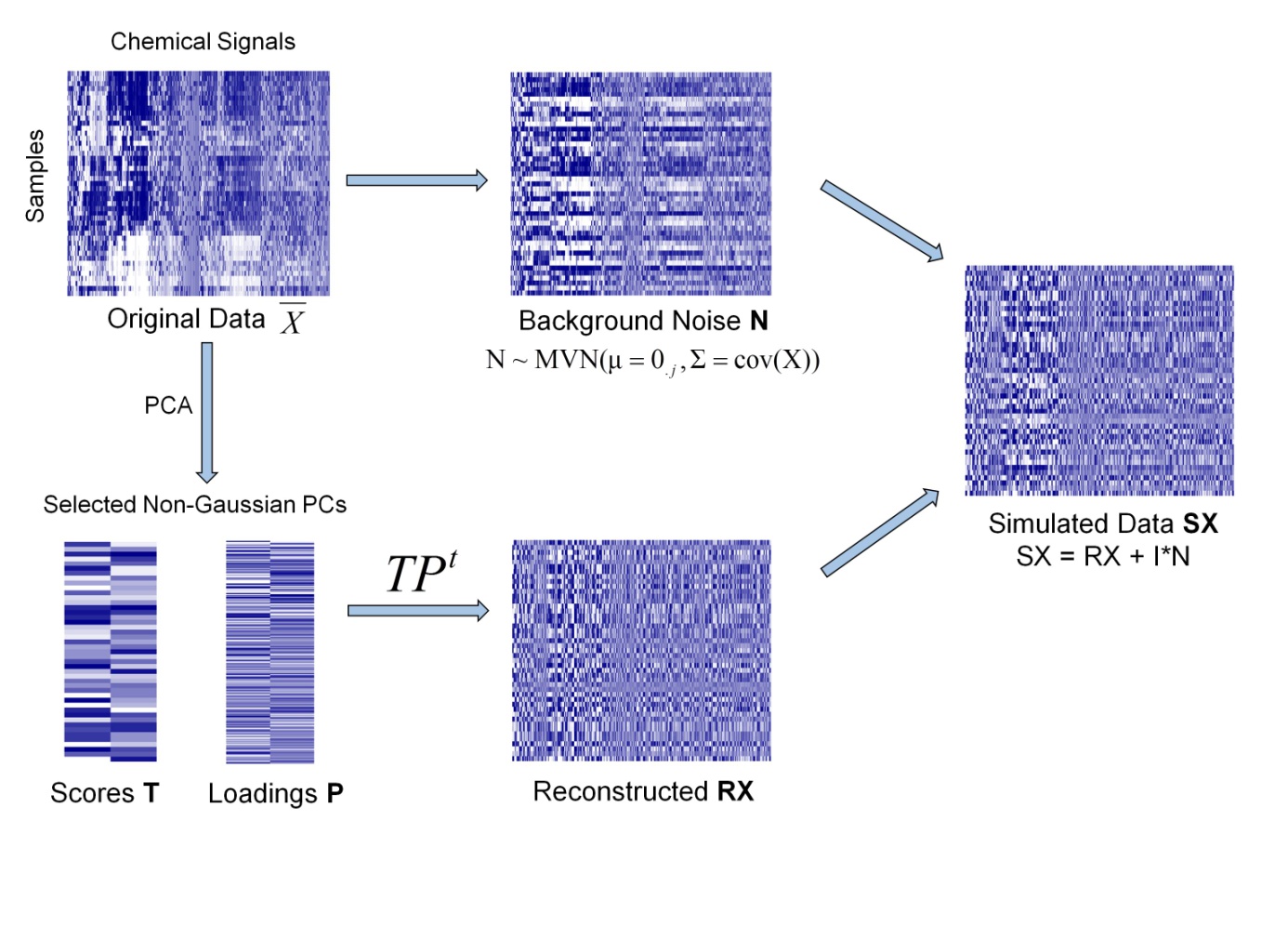
**Figure S1. Generation of simulated data**


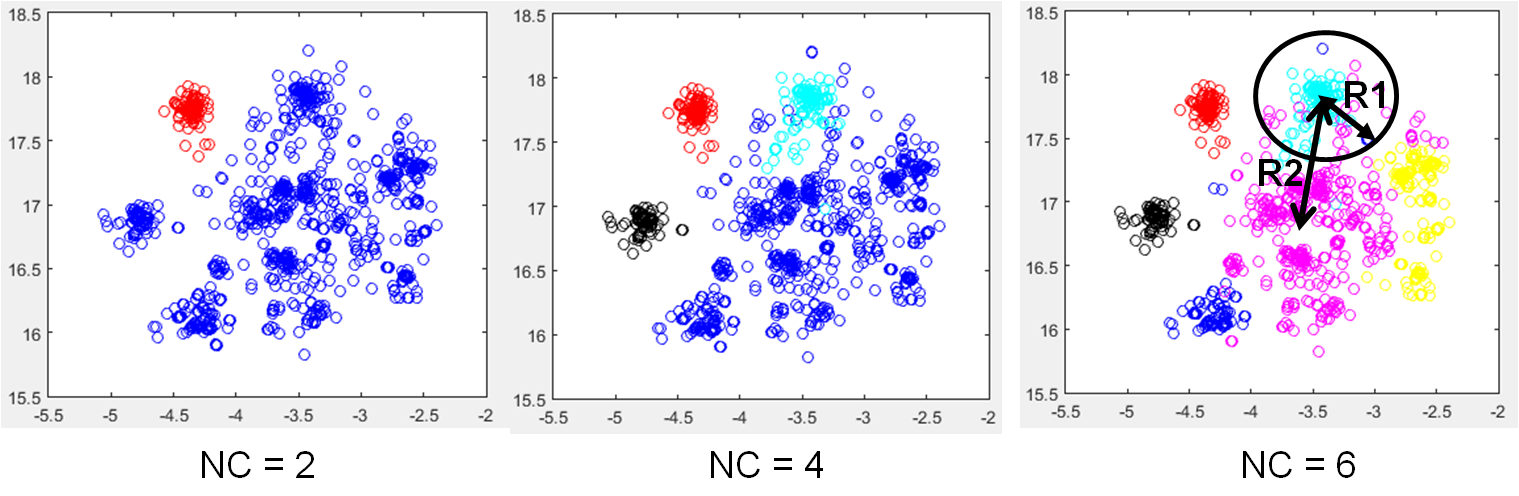


**Figure S2. Hierarchical clusters on 2D space**

**
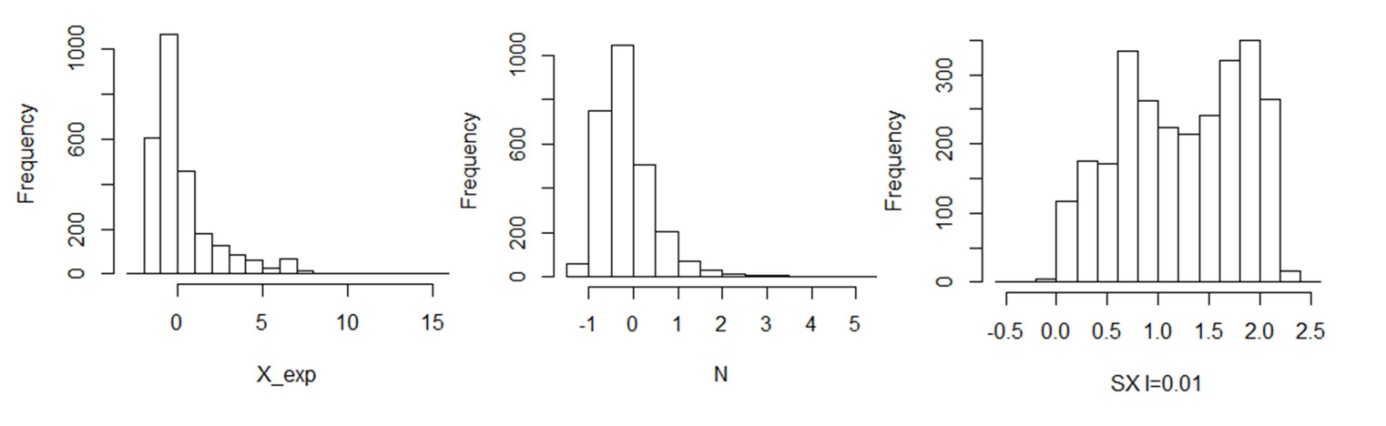
**

**Figure S3. Kurtosis distribution of all variables (masses)**


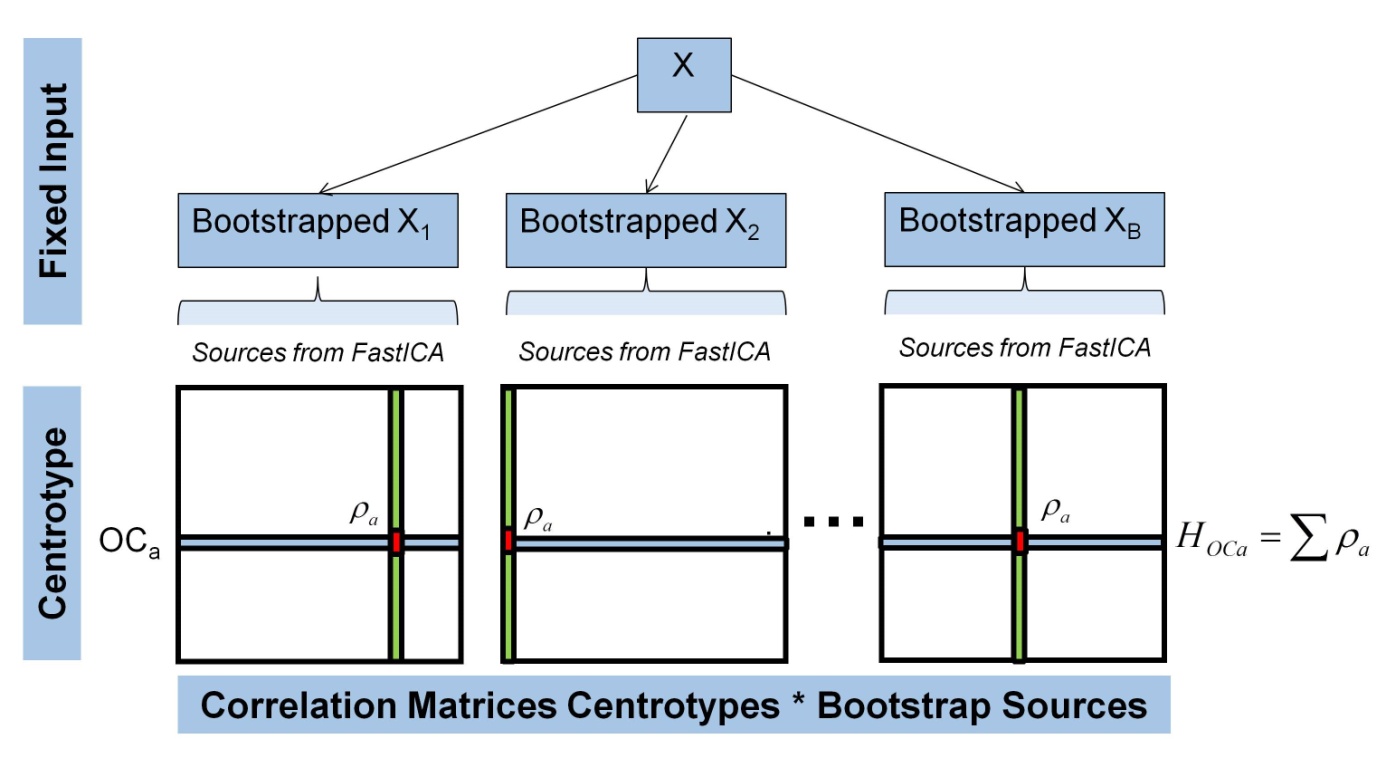


**Figure S4. Illustration for bootstrap scores**


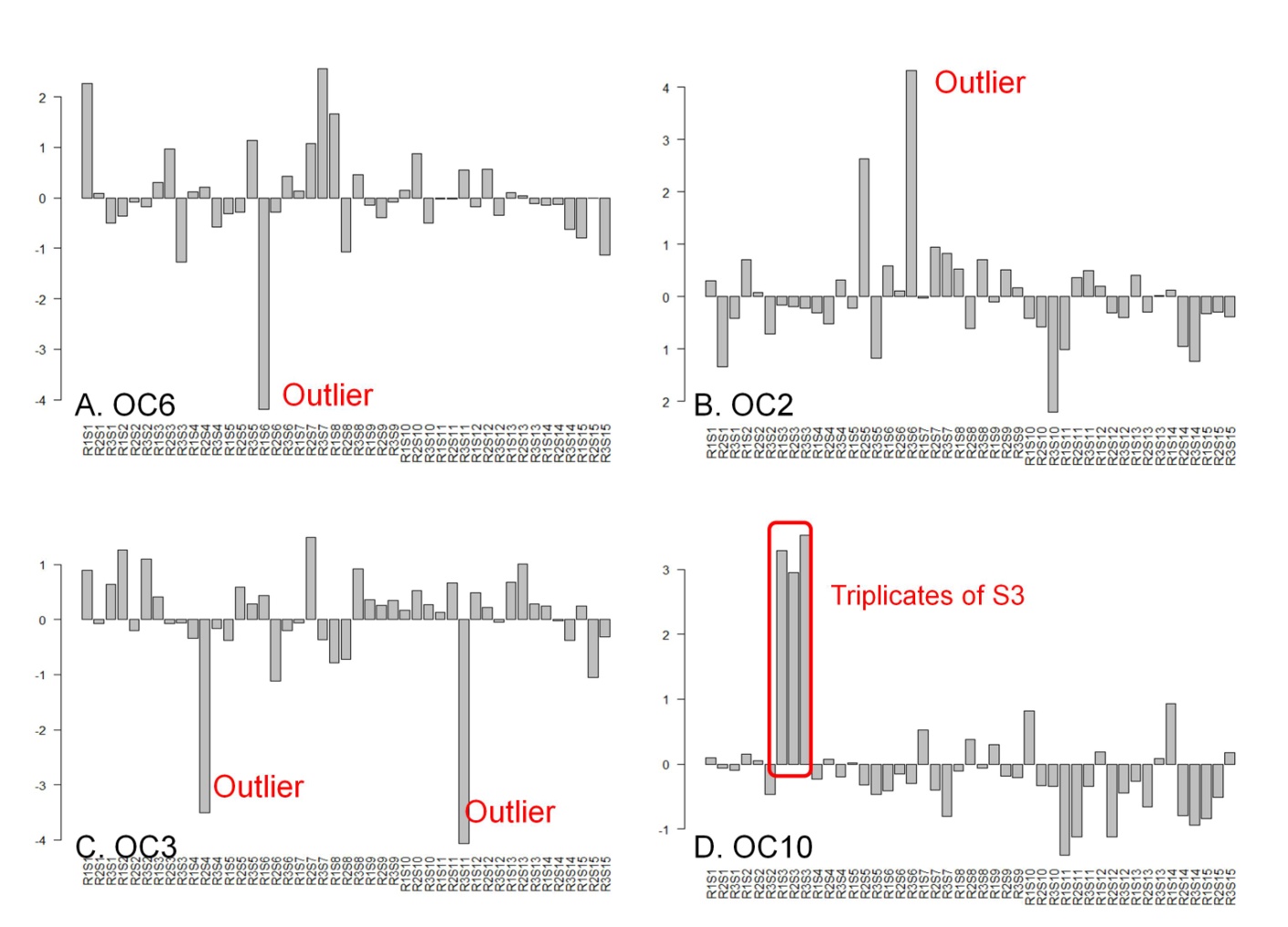


**Figure S5. Scores of samples on some centrotypes**
