# Supplementary material for: MetICA: independent component analysis for high-resolution mass-spectrometry based non-targeted metabolomics
Source: BMC Bioinformatics. 2016 Mar 2;17:114. doi: 10.1186/s12859-016-0970-4 (PMC4776428; doi:10.1186/s12859-016-0970-4)
Supplement: Additional file 3: — Evaluation of MetICA on lower resolution metabolomic data. Additional text and figures are provided to illustrate the application of MetICA on lower resolution LC-MS data. (DOCX 280 kb) [file 12859_2016_970_MOESM3_ESM.docx]

**Evaluation of *MetICA* on lower resolution metabolomic data**

Although not described in the main text, the algorithm *MetICA* was tested on several metabolic profiling datasets generated from TOF-MS (Synapt HDMS ao-Q-TOF, Waters, Milford, MA) coupled to the ACQUITIY UPLC system (Waters, Milford, MA). The TOF-MS was less sensitive and less accurate than FT-ICR-MS. As an example, same samples (45 wines fermented by 15 yeast strains) were analyzed on LC-TOF-MS in (-)ESI mode. The dataset generated contained 11548 LC-MS features. Each LC-MS feature was a pair of retention time (RT) and m/z. The test dataset 'Yeast-Experimental-LC-MS.dat' can be found at https://github.com/daniellyz/MetICA.

*MetICA* was applied on the raw dataset by keeping 90% of total variance, thus 27 independent components were extracted from each FastICA run. From 400 FastICA runs, 400 * 27 = 10800 estimated sources were clustered using CCA algorithm. Based on the distribution of estimated sources, we have chosen 13 partitions to generate compact and well-separated clusters (Figure A). The centrotypes of these clusters, or MetICA components, were evaluated by comparing to 50 bootstrapped data in 100 MetICA runs. The bootstrapping score H has explicitly ranked the MetICA components according to their statistical reliability (Figure B). The consistency between different algorithm runs (the spread of box plots) indicated the sensitivity of our evaluation.

In summary, for both high resolution (FT-ICR-MS) data and low resolution (LC-MS) data, MetICA was able to reduce FastICA estimate errors by clustering estimated sources. It was also able to select the most reliable components by bootstrapping. The sensitivity of these evaluations has been proved in various datasets independent from data quality.


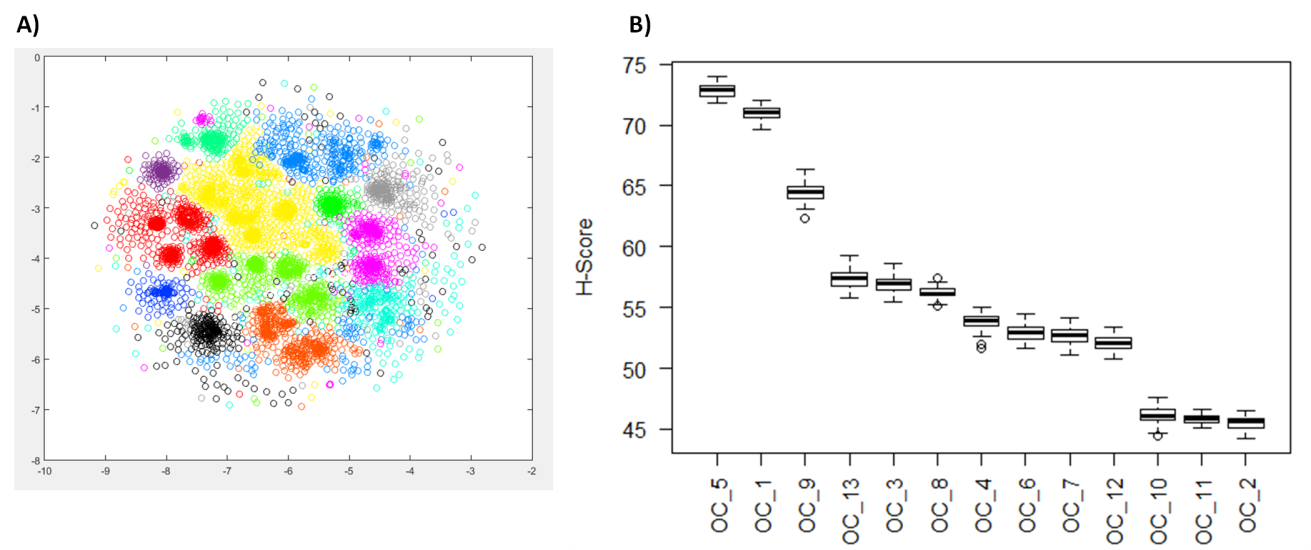


**Figure A** Distribution of 10800 MetICA sources (for three background noise levels) when projected on a 2D CCA space.

**Figure B** The distribution of H estimate of each MetICA component by box plot. The components were sorted by their median.

We note also that ultrahigh resolution mass spectrometry, such as FT-ICR-MS, although without chromatographic separation, generated more than 10 000 features. In non-targeted metabolomics, various feature filtration techniques could be applied before statistical analysis to avoid heavy computational load. For instance, we have applied the software *Netcalc* to reduce the number of features from 20 000 to 2 700 (more details in Metabolomics data acquisition and pre-treatment in the Method section).Our evaluation on raw LC-MS data has shown the ability of MetICA to extract sensitively information from more than 10 000 features despite a longer execution time. Moreover, *Netcalc*-based feature filtration could be applied to lower-resolution mass spectrometry without losing annotation confidence [1]. In reality, we would hardly need to perform MetICA on data matrices that contain more than 10 000 features. Therefore heavy computational load was not problematic for either lower or higher resolution MS-based metabolomics data.

[1] Forcisi S, Moritz F, Lucio M, Lehmann R, Stefan N, Schmitt-Kopplin P: **Solutions for low and high accuracy mass spectrometric data matching: a data-driven annotation strategy in nontargeted metabolomics**. *Anal Chem* 2015, **87**:8917–8924.
